# Effects of dietary fat manipulation on cognition in mice and rats: protocol for a systematic review and meta-analysis

**DOI:** 10.1136/bmjos-2020-100108

**Published:** 2020-11-18

**Authors:** Fiona J Ramage, Alexander S Clewlow, Lynda M Williams, Malcolm R Macleod, Rosamund F Langston

**Affiliations:** 1 Department of Systems Medicine, University of Dundee, School of Medicine, Dundee, UK; 2 GKT School of Medical Education, King's College London, Faculty of Life Sciences and Medicine, London, UK; 3 The Rowett Institute, University of Aberdeen Rowett Institute of Nutrition and Health, Aberdeen, UK; 4 Centre for Clinical Brain Sciences, The University of Edinburgh, Edinburgh Medical School, Edinburgh, Scotland, UK

## Abstract

**Introduction and objective:**

The Western diet that comprises high levels of long-chain saturated fats and sugar is associated not only with metabolic disorders such as obesity and type 2 diabetes but also has been recently linked to brain changes and cognitive dysfunction. However, in animal studies, reported effects are variable, and the mechanisms underlying these effects are unclear. In the proposed review, we aim to summarise the diverse evidence of the effects of so-called ‘high-fat’ and ketogenic diets on behavioural measures of cognition in postweaning mice and rats, relative to animals on standard diets and to determine potential underlying mechanisms of high-fat diet-induced effects.

**Search strategy:**

A comprehensive search strategy was designed to retrieve studies reporting use of a high-fat or ketogenic diet in postweaning mice and rats that included cognitive assessments. Three databases (Medline, SCOPUS and Web of Science) were searched and 4487 unique references were retrieved.

**Screening and annotation:**

Studies were screened for inclusion by two independent reviewers, with 330 studies retained for analysis. Characteristics of disease model choice, experimental design, intervention use and outcome assessment are to be extracted using the Systematic Review Facility (http://syrf.org.uk/) tool. Studies will be assessed for study quality and risk of bias and confidence of mechanistic involvement.

**Data management and reporting:**

For cognitive outcomes, effect sizes will be calculated using normalised mean difference and summarised using a random effects model. The contribution of potential sources of heterogeneity to the observed effects of diet on cognition will be assessed using multivariable meta-regression, with partitioning of heterogeneity as a sensitivity analysis. A preliminary version of this protocol was published on 9 April 2019 on the Collaborative Approach to Meta-Analysis and Review of Animal Data from Experimental Studies website (http://www.dcn.ed.ac.uk/camarades/research.html%23protocols).

**Ethics and dissemination:**

No ethical approval is required as there are no subjects in the proposed study.

**Strengths and limitations of this study**

- This systematic review will comprehensively collect and analyse available evidence relating to the effects of dietary fats on cognition as well as summarising proposed underlying mechanisms.
- Due to differences in study findings, proposed mechanisms as well as experimental design, a comprehensive summary will be of great benefit to the field.
- This review will have a strong emphasis on evaluating study design, risk of bias and strength of evidence provided by included studies.
- An earlier version of the protocol for this study was registered with the Collaborative Approach to Meta-Analysis and Review of Animal Data from Experimental Studies facility.
- A large degree of heterogeneity in diets, animal models and experimental design used in included studies may limit our ability to summarise the overall effects of diet on cognition and potential underlying mechanisms.

## Introduction

The incidence of diseases associated with lifestyle factors such as diet has been steadily increasing over the last few decades and poses a significant global healthcare burden.^1 2^ Western diets, typically high in long-chain saturated fats and refined carbohydrates, are linked with the development of metabolic dysfunction characterised by obesity, hypertension and insulin resistance.^3 4^ This is termed the metabolic syndrome and places individuals at increased risk of disorders including type 2 diabetes mellitus and cardiovascular disease.^5–7^ Recent evidence suggests that consumption of a Western diet may also be associated with cognitive impairment and changes to brain structure and function. Impaired cognition appears to be more common, and more pronounced, in people with obesity^8 9^ or type 2 diabetes mellitus.^10^ ^11 12^ The consumption of a Western diet has been associated with the development of dementia including Alzheimer’s disease,^13 14^ and metabolic syndrome itself is an identified risk factor for the condition.^15^ In the Baltimore Longitudinal Study of Ageing, cognitively normal individuals with metabolic syndrome did not have increased cerebral amyloid (determined using Pittsburgh compound B (PiB) Positron Emission Tomography (PET) neuroimaging) at baseline when compared with patients without the metabolic syndrome, but the rate of accumulation of cerebral amyloid in the following 2.6 years was higher in PiB PET-positive persons with metabolic syndrome than in PiB positive persons without metabolic syndrome; and these changes were more pronounced in brain regions associated with the early stages of Alzheimer’s disease.^16^ However, studies in the Lothian Birth Cohort^17^ suggest that the association between body mass index and impaired cognition in adults could be largely explained by differences in childhood IQ and socioeconomic factors.

It is therefore not clear whether metabolic syndrome causes an increased incidence of cognitive decline in later life and a more rapid progression of a pre-existing cognitive decline; or whether metabolic syndrome and cognitive decline are independent but share risk factors. Because of the increasing numbers of those affected by metabolic disorders and cognitive impairment,^1 18^ understanding dietary effects on cognition and the mechanisms that subserve these effects is of critical importance for human health.

Most preclinical studies have focused on the effects of dietary fat and carbohydrate, alone or in combination, and this systematic review will particularly focus on describing the effects of dietary fat on cognitive function in mice and rats and the potential mechanisms of any effect. The scope of our systematic review is restricted to the study of mice and rats, to limit between-study heterogeneity. Many rodent studies suggest that the consumption of a diet high in fat (a ‘high-fat diet’) and refined sugars leads to cognitive deficits, confirming effects observed in human studies. Diet-induced cognitive deficits in rodent models have been reported in various learning and memory tasks,^19–23^ though reported effects often vary between studies.^24^ Preclinical research additionally supplies more detailed information about potential central mechanisms involved in these effects. Rodent studies have shown that consumption of a high-fat diet associated with cognitive impairment is linked to brain inflammation, causing increased levels of proinflammatory cytokines and immune cell activation.^19 25 26^ Oxidative stress has also been shown to occur in the brains of high-fat-fed rodents and cognitive impairment to be alleviated by the administration of antioxidant treatments.^27 28^ Moreover, high-fat diet-induced cognitive impairments have been linked to deficits in insulin signalling and brain insulin resistance^29 30^ as well as changes to synaptic plasticity,^31^ including alterations to neurotrophic factors such as Brain-Derived Neurotrophic Factor (BDNF).^32^

Interpretation of observed effects of high-fat diets on cognition or potential underlying mechanisms may not be straightforward if there is heterogeneity in experimental designs and in the definition and description of the dietary interventions. Various terms are used to describe diets with high(er) levels of fat including ‘high-fat diets’, ‘Western diets’, ‘high-fat-sugar diets’, ‘high-energy diets’ or ‘cafeteria diets’. The exact composition of these diets and the control diets used, for instance, the content and types of fats and carbohydrates, may be variable and not completely defined. It is not known whether effects vary when using different species, strains and ages of experimental animals or the impact of varying key experimental parameters such as duration of diet exposure and type of behaviour assessments employed.^24 33^ Finally, in other fields, low levels of measures to reduce the risks of bias have been reported in in vivo research,^34 35^ and that lower reporting is associated with larger estimates of biological effect. The extent to which in vivo research on the impact of dietary fat intake on cognition might be similarly affected is not known.

Of particular interest are studies of the effect of high-fat diets with very low levels of carbohydrates. These are referred to as ketogenic diets as they induce the production of ketone bodies from fat (ketogenesis) as an alternative energy source to glucose^36^ and have been suggested as therapies for a number of conditions.^37 38^ Studies variously suggest that in animals, ketogenic diets are neuroprotective and boost cognition^39^ or are detrimental to brain function.^40^

Our understanding of the effects of dietary fat on brain function would be enhanced by a comprehensive summary of in vivo research. Here we present a protocol for a systematic review and meta-analysis of the effects of diet composition, particularly fat, on cognitive function in mice and rats, and of the available evidence regarding potential underlying mechanisms. We will evaluate heterogeneity in study designs and dietary manipulations and assess reporting of measures to reduce the risk of bias as well as assessing the presence of possible publication bias.

## Methods

An earlier version of this protocol has been published (9 April 2019) online.^41^

### Research question and search strategy

This systematic review aims to describe the reported effects of manipulations of dietary fat on cognition in mice and rats and to review and assess the evidence for the mechanisms of these effects. The effects of high-fat diets on cognition will be determined by extracting outcome data in behavioural tests of cognition. Detailed study characteristics (animal and diet characteristics, intervention, experimental design, outcome assessment) will be collected and analysed, and their impact on outcome measures will be assessed. We also aim to assess study quality and measures to limit risk of bias and to evaluate the impact of these on reported results. Specific questions are summarised in box 1.

Box 1 **Summary of research questions to be addressed in the systematic review**

What are the effects of manipulations of dietary fat on cognition in otherwise healthy mice and rats?

What are the possible mechanisms underlying these effects?

If there is heterogeneity in reported findings, can this be accounted for by identifiable differences between experiments?

What is the heterogeneity in experimental characteristics and the quality of their reporting?

What is the prevalence of reporting of measures to reduce risks of bias?

Does reporting of measures to reduce the risk of bias explain any of the observed heterogeneity?

Is there evidence of publication bias?

What is the strength of evidence for causal relationships between individual proposed mechanisms of action and observed cognitive change?

The primary research questions were formulated as a PICO (Patient - Intervention - Comparator - Outcome) framework^42^:

Population of interest: postweaning mice and rats

Intervention: any manipulation of dietary fat

Control population: animals fed a standard diet

Outcome measures: behavioural measures of cognitive performance.

We designed a search strategy for Medline via PubMed, SCOPUS and Web of Science using published guidelines^43^ (table 1).

**Table 1:** Keywords used in comprehensive search in Medline (PubMed), SCOPUS and Web of Science electronic databases

Database	Search terms	
Medline	“High-fat diet” [MeSH] OR “high-fat diet” [tiab] OR “HFD” [tiab] OR “western diet” [tiab] OR “cafeteria diet” [tiab] OR “obesogenic diet” [tiab] OR “diet induced obesity” [tiab] OR “high fat-sugar diet” [tiab] OR “high fat high sucrose” [tiab] OR “high fat high fructose” [tiab] OR “long-chain saturated fats” [tiab] OR “high-lipid diet” [tiab] OR “high-fat fed” [tiab] OR “HFHS” [tiab] OR “high-energy diet” [tiab] OR “ketogenic diet” [MeSH] OR “ketogenic diet” [tiab] OR “very low carbohydrate diet” [tiab] OR “VLCD” [tiab] OR “low carbohydrate diet” [tiab] OR “carbohydrate-restricted diet” [tiab] OR “restricted carbohydrate diet” [tiab] OR “low-carbohydrate high-fat diet” [tiab] OR “high-fat low-carbohydrate diet” [tiab)	**#1**
	mice [MeSH] OR mice [tiab] mouse [tiab] OR rat [MeSH] OR rat [tiab] OR rats [tiab] OR murine [tiab] OR mus [tiab] OR rattus [tiab] OR rodent [tiab)	**#2**
	(Cognitive [tiab] OR executive [tiab)) AND (function [tiab] OR dysfunction [tiab] OR performance [tiab] OR deficits [tiab] OR deficit [tiab] OR rigidity [tiab] OR flexibility [tiab))) OR behaviour [tiab] OR behavior [tiab] OR behavioural [tiab] OR behavioral [tiab] OR memory [tiab] OR learning [tiab] OR “task performance” [tiab] OR cognition [tiab] OR “neural activity” [tiab] OR “brain activity” [tiab] OR “neuronal activity” [tiab] OR “neuro-behavioural” [tiab] OR neurological [tiab] OR ((spatial [tiab] OR episodic [tiab] OR executive [tiab] OR recognition [tiab)) AND (memory [tiab] OR task [tiab] OR function [tiab))) OR “synaptic plasticity” [tiab] OR neuroplasticity [tiab] OR “neural plasticity” [tiab] OR “long-term potentiation” [tiab] OR “long-term depression” [tiab] OR LTP [tiab]	**#3**
SCOPUS and Web of Science	“high fat diet” OR “HFD” OR “western diet” OR “cafeteria diet” OR “obesogenic diet” OR “diet induced obesity” OR “high fat sugar diet” OR “high fat high sucrose” OR “high fat high fructose” OR “long chain saturated fats” OR “high lipid diet” OR “HFHS” OR “high energy diet” OR “ketogenic diet” OR “ketogenic diet” OR “very low carbohydrate diet” OR “VLCD” OR “low carbohydrate diet” OR “carbohydrate restricted diet” OR “restricted carbohydrate diet” OR “low carbohydrate high-fat diet” OR “high fat low carbohydrate diet”	**#1**
	mice OR mouse OR rat OR rat OR rats OR murine OR mus OR rattus OR rodent	**#2**
	((cognitive OR executive) AND (function OR dysfunction OR performance OR deficits OR deficit OR rigidity OR flexibility)) OR behaviour OR behavior OR behavioural OR behavioral OR memory OR learning OR “task performance” OR cognition OR “neural activity” OR “brain activity” OR “neuronal activity” OR “neuro-behavioural” OR neurological OR ((spatial OR episodic OR executive OR recognition) AND (memory OR task OR function)) OR “synaptic plasticity” OR neuroplasticity OR “neural plasticity” OR “long-term potentiation” OR “long-term depression” OR ltp	**#3**

### Study selection and inclusion/exclusion criteria

All primary research articles were included, with no date restrictions, which described the effect of manipulation of dietary fat on cognitive outcomes in rats and mice with or without use of an intervention intended to moderate cognition, where the dietary manipulation occurred after weaning. Exclusion criteria were studies in languages other than English, where full text was not available (despite attempts to contact the author); review articles, systematic reviews, book chapters and conference abstracts; those involving animals with comorbidities other than those induced by diet (including transgenic animals, unless the impact of transgenesis on the effect of diet was the objective of the study), where the dietary intervention was maternal or paternal or occurred preweaning and where the only reported outcomes were measures of anxiety and depression, locomotor activity, feeding behaviour, addiction or social behaviours.

The first version of the protocol^41^ proposed to include studies where the dietary intervention started when animals were at least 12-week old and had finished the period of rapid growth. However, during the screening process (see below), it was found that many studies used younger animals, and on reflection, it was considered that dietary modification in early life, but after weaning, was of biological importance and of interest for the review; the inclusion criteria were therefore amended accordingly.

Studies were screened in two stages. First, two independent reviewers (FJR and ASC) screened studies based on title and abstract, and full texts of remaining articles were retrieved. The inclusion and exclusion criteria were then reapplied, by the same reviewers, with differences resolved in discussion with a third reviewer (RFL). Screening was conducted manually using EndNote V.X9.

### Collection of study characteristics

Qualitative and quantitative data will be extracted using the Systematic Review and Meta-analysis Facility (SyRF) platform (http://syrf.org.uk/). Included studies will be imported to the SyRF platform (app.syrf.org.uk) for annotation and outcome data extraction. Annotations include characteristics of (1) animals (species, strain, age, weight, sex), (2) control and experimental diets (fat and carbohydrate content, detailed macronutrient composition, feeding method), (3) intervention (name, dose, administration route, duration/schedule of administration, type/category, proposed mechanism of action), (4) of experimental designs (age at diet introduction, duration of diet exposure, timing of intervention administration relative to diet), (5) cognitive outcomes measured (behaviour task used, timing of outcome assessment relative to diet/intervention administration, exact description of outcome assessment method) and (6) details of metabolic (eg, body weight and composition, insulin sensitivity) and neuropathological (eg, changes to brain markers or neuronal activity) outcomes reported.

### Study quality appraisal and risk of bias

Study quality will be evaluated using a checklist based on the Collaborative Approach to Meta-Analysis and Review of Animal Data from Experimental Studies study quality checklist,^44^ being

- Publication in a peer-reviewed journal.
- Declaration of potential conflicts of interest.
- Statement of compliance with animal welfare regulations.
- Appropriately detailed reporting of basic animal and housing characteristics and
- Appropriate statistical power/sample size calculations.

In addition, domain-specific features will be considered, being

- Whether the study measures food intake and reports this for each group (to ensure that effects on outcome are not due to differences in food intake rather than diet composition).
- Reporting of any additional experimental manipulations that could have interfered with the measurement of primary and secondary outcomes (such as the use of fasting for intraperitoneal glucose or insulin tolerance tests or the use of food reward-based or stressful behavioural tests that may confound the effect of diet).
- Evaluation of possible confounding factors for cognitive outcome measures (eg, total object exploration time or swimming speed).
- The nature of the control intervention (adequately matched to the intervention, rather than the absence of intervention).
- The nature of the control diet and whether it is adequately matched to the experimental diet.
- Appropriate statistical analysis and
- Whether any interventions might have interfered with the establishment of the disease model (if they were administered prior to the establishment of the high-fat diet model, ie, prior to animals having consumed experimental diets for 12 weeks).

Risks of bias will be assessed using a modified version of the risk of bias tool proposed by SYRCLE (SYstematic Review Center for Laboratory animal Experimentation)

- Selection bias
  - Were animals randomised to diet/treatment groups?
  - Was this group allocation adequately concealed?
  - Were baseline differences between groups assessed?
    - If differences were present, were adequate adjustments made to account for this?
    - Could these have influenced outcome?
- Performance bias
  - Was allocation to home cage randomised?
  - Were animal technicians blinded to group allocation?
  - Were researchers blinded to group allocation?
- Detection bias
  - Where not every animal had every outcome measured, were animals selected randomly for outcome assessment?
  - Was the outcome assessor blinded to diet/treatment group?
- Attrition bias
  - Were any animals excluded from the analysis (attrition)?
  - Were reasons for exclusion adequately described?
- Reporting bias
  - Was there any evidence to suggest selective outcome reporting?
- Other
  - Is the unit of analysis appropriate (ie, is there evidence of pseudoreplication?).

### Certainty of evidence

A secondary objective is to evaluate the possible mechanisms of diet-induced cognitive change. Mechanisms potentially involved will be inferred based on effectiveness of different therapeutic interventions on cognitive performance and the effects of dietary manipulation or interventions on secondary neuropathological or metabolic outcomes. Different studies will provide evidence of different strength, and confidence in the claims made as well as the overall quality of provided evidence, will be evaluated using a modified GRADE^45^ (Grading of Recommendations, Assessment, Development and Evaluation) approach. Specifically, the following points will be considered:

- Was the intervention tested in a control group and did it affect primary or secondary outcomes in this group?
- If the study used an intervention did the study confirm the effect of the intervention on the proposed target or mechanism of action?
- Were any secondary neuropathological or metabolic outcomes compared for statistical correlation with cognitive outcome measures?
- Does the study present any in vitro or mechanistic data relevant to the hypothesis?
- Does the study refer to or present any replications (technical, biological, conceptual) of the same experiment?
- Was the intervention tested at several different doses and was there a dose-dependent effect?

Additionally, confidence in results will be increased for studies with large magnitude of effect. Confidence will be decreased by low study quality, high risks of bias, inconsistency of findings and imprecision (large confidence intervals). Given the expected heterogeneity in the circumstances of experimental evaluation, heterogeneity of observed effects will not reduce confidence in the findings presented.

### Extraction of outcome data

Outcome data will be extracted for behavioural tests measuring cognitive ability. This will include tests of learning and spatial (eg, Morris water maze, Y-maze) and non-spatial (eg, novel object recognition) memory as well as tests of executive function. The effects of diet on measures of locomotor activity, anxiety and depression behaviours, social behaviours, motivation or feeding behaviours are not within the scope of this review. Non-cognitive components of eligible cognitive tasks (eg, swim speed for Morris water maze, total object exploration time for novel object recognition) will also not be quantitatively assessed. The effects of diet on cognition will be determined by comparisons in task performance between high-fat and standard diet groups and the effects of interventions on cognition determined by comparing intervention and control groups in animals fed a high-fat diet.

For each outcome reported, the number of animals used in the experiment, the outcome in each group (mean or median) and the reported variance (SD or SE of the mean) will be recorded. Where cognitive outcomes have multiple components, each component will be recorded, noting that these have been measured in the same cohort of animals at the same timepoint. Quantitative data will be extracted from text or data tables or from figures using either a digital ruler tool or the embedded SyRF Graph2Data tool if available. Where all data cannot be ascertained, authors will be contacted for further information. Where sufficient data are not available, the data point will be excluded from analysis. Missing values will not be imputed.

### Quantitative analysis

(1) The effects of dietary manipulation on cognition and (2) the effect of experimental interventions in moderating the effects of dietary fat manipulation on cognition will be considered separately. Standardised mean difference estimates of effect size^46^ are associated with reduced meta-analytical power when group sizes are small^47^ but normalised mean difference (NMD) estimates of effect size are not always feasible. Therefore, NMD effect size estimates will be used unless this would lead to a loss of information of 30% or more (ie, unless NMD estimates are not feasible for at least 70% of effect sizes). Findings will be summarised using random effects meta-analysis performed using the SyRF R-shiny app using the restricted maximum likelihood estimator of tau, presenting central estimates and 95%CIs.^46^

The contribution of potential sources of heterogeneity in the observed effects of diets on cognitive outcome measures will be assessed using multivariable meta-regression. The independent variables to be tested in this prespecified analysis are disease model characteristics (age, sex, dietary fat and carbohydrate content and macronutrient composition); experimental design parameters (behaviour task used, duration of diet exposure); intervention characteristics (type, proposed mechanism of action—ie, anti-inflammatory) and study quality and risk of bias measures. Given the limited statistical power of meta-regression, differences between groups will also be reported, as a sensitivity analysis, using partitioning of heterogeneity with appropriate Bonferroni correction to adjust for multiple comparisons in each of disease model characteristics, experimental design parameters, intervention characteristics and study quality and risk of bias measures. Secondary (neuropathological or metabolic) outcomes will be evaluated using the same approach.

Finally, the possible presence of publication bias will be evaluated using funnel plotting, Egger regression and ‘trim-and-fill’ analysis.

### Progress to date

**Figure 1:**
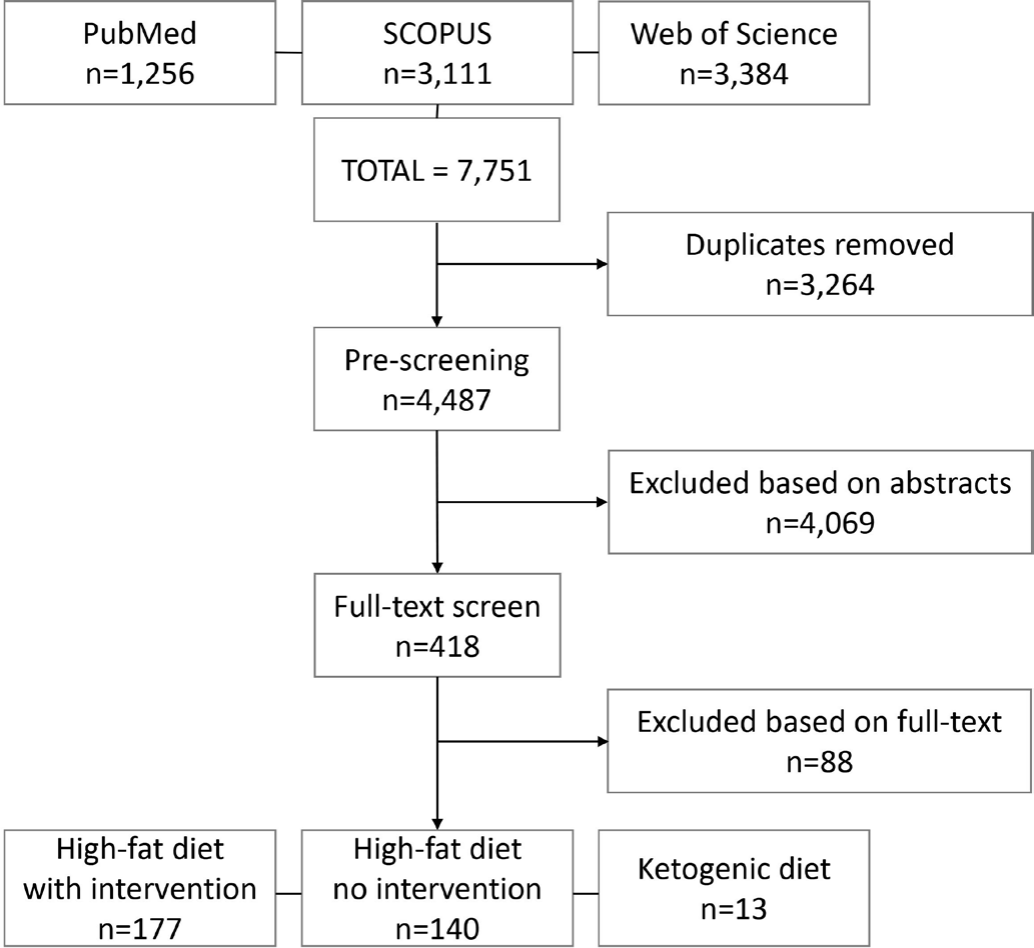
Preferred Reporting Items for Systematic Reviews and Meta-Analyses flow diagram^55^ of studies screened for inclusion in the systematic review and meta-analysis.

Search terms were applied on 9 April 2019 and updated on 12 February 2020. A total of 7751 studies were retrieved, and after removal of duplicates, 4487 articles were screened on the basis of title and abstract. In that 418 progressed to the full-text screening phase and 330 articles were retained to be included in the review (177 high-fat diet intervention studies, 140 high-fat diet without intervention and 13 ketogenic diet studies) (figure 1).

## Discussion

Preclinical systematic reviews are useful tools for synthesising large numbers of animal studies that provide conflicting results. These reviews can address inconsistencies, inform the design and content of future preclinical research as well the design of human clinical studies.^48^ Animal studies can be heterogenous, employing different models, ages and species or strains. Diets may also be diverse, consisting of variable proportions and types of fats, carbohydrates and protein. Different behavioural tasks and methods of outcome assessment to measure cognitive performance may also be used.^24 33^ Summarising available evidence and reaching robust conclusions of the effects of diet composition, particularly fat, on cognition and the underlying mechanisms for any effects is therefore challenging and has not to date been systematically addressed in reviews of the field.

The proposed systematic review was designed to comprehensively describe the effects of dietary fat manipulation on cognition and evaluate proposed mechanisms behind these effects. By collecting details of model selection and experimental design and assessing how these are associated with differences in cognitive outcome, we aim to understand better the observed heterogeneity in reported effects of dietary fat on cognition. For instance, studies have suggested that responses to high-fat diets may depend on the age^49^ and sex^50^ of experimental animals and have shown different patterns in cognitive impairment based on behaviour task used (eg, spatial vs non-spatial) and duration of exposure to diet.^51–53^ Additionally, by reviewing studies using high-fat but very low-carbohydrate ketogenic diets, we hope to further understand the separate roles of fat and carbohydrate diet components in inducing cognitive changes. Confidence in the attribution of mechanistic pathways will be evaluated using a GRADE approach^45^ method of evidence assessment.

Limited reporting of study design and procedures to minimise bias has been shown to impact outcome effect estimates in preclinical research^44 48^ and is therefore an important consideration in assessing the validity of any evidence provided. This review will adapt existing tools^44 54^ to assess study quality, risk of bias and certainty of evidence provided in this field. We hope therefore to systematically assess methodological strengths and weaknesses of included studies and to determine whether these factors impact overall results as well as critically evaluating the likelihood of mechanistic contributions.
